# Physiological Effects of Exercise upon the Human Body

**Published:** 1863-11

**Authors:** 


The Physiological Effects of Exercise upon the Human Body.—The investigations of Speck, upon the influence of exercise on the human body, embrace two new series of experiments, which the author made upon himself, and those which he made on two persons, aged 23 years, and one upon a young man of 19 years. His conclusions are as follows:—

Corporeal exercise has, as its consequence, the diminution of the weight of the body. Since there is but a small change, unless the weight of the body be at once determined, after the exercise has been taken, there might be an error in the estimate. The diminution in the weight ceases cotemporaneously with the cessation of the muscular activity;—in case there should continue any excretory action after the muscles are at rest, this should not be included in the estimate of the loss. The researches of Speck afford no positive evidence whether or no moderate muscular exercise favors a reparation of the loss sustained.

The use of water during corporeal exercise appears to act differently than during inaction: during rest, the use of water diminishes the weight of the body; on the contrary, during action, the drinking of water is accompanied by an augmentation of the body's weight, the water being probably retained to compensate for the loss of fluids, which otherwise ensues.

Muscular exertion constantly diminishes the whole quantity of urine which is excreted; during action, the quantity may be reduced to two-thirds, or even one-half of the normal amount. The cause of the diminution of the quantity of the urine during action, is, that there is, during exercise, an increased cutaneous transpiration;—hence, during exercise, the urinary excretion contains more solid materials than usual, so that, in this respect, muscular activity becomes an important agent in promoting renal elimination.

During labor, the skin and the lungs become the main excretory outlets; the excretory processes are more active at the close of the afternoon than during the forenoon. During active exercise, the perspiration may be increased to three-fold its usual amount;—after the exercise has ceased, the perspiration is rapidly reduced, or may wholly cease.

The fæcal evacuations are, as a rule, less during exercise than during repose. Food of the same kind appears to be alike digested during repose or muscular activity;—it is probable that the less weight of the excrements, during exercise, is dependent upon the want of the aqueous element. Intestinal peristalsis occurs more slowly during violent exercise.

There has been observed no perceptible alteration in regard to the quantity of urea which is eliminated during active bodily exercise. * * * Physical exercise increases, to a great extent, the amount of uric acid that is discharged;—indeed, it is augmented beyond any other urinary ingredient; for example, it is augmented to double or even three-fold its normal amount. As the use of the water diminishes the relative amount of the uric acid in urine, so on the contrary, free perspiration increases it.

Since the perspired matter contains, in considerable quantity, chloride of sodium, hence, when the cutaneous transpiration is profuse, the quantity of chloride of sodium in the urine is lessened.

All researches made indicate a considerable increase of sulphuric acid in the urine during exercise; this augmentation continues for some time after the exercise has been discontinued. The perspiration seems to remove little or no sulphuric acid.

Phosphoric acid is also considerably augmented in the urine, both during and after exercise; the quantity of this acid which is excreted during free perspiration appears less than that during diminished perspiration, hence the inference that there is a portion of phosphoric acid excreted also through the skin. * * *

The quantity of the air which traverses the lungs is gradually increased from morning until evening, so that in the evening from 8 to 9 o'clock, the maximum is attained. Exercise very notably augments this amount; the augmentation is manifest, even when the number of respirations remains the same. The quantity of carbonic acid which is eliminated is more increased than the amount of air;—for example, during gentle exercise the elimination of this gas becomes double the normal standard, and during violent exercise the quantity of carbonic acid excreted is augmented to three-fold the usual amount.

The heat of the organism is somewhat increased by exercise, though, soon after the exercise has ceased, the temperature rapidly sinks even to a point below the normal degree. The production of the perspiration appears to be accompanied with an increase of bodily heat. The lowest temperature of the body is in the morning, the highest at mid-day, and in the evening there occurs a diminution again.—*Translated from Canstatt's Jahresbericht.*

